# Preserved Consciousness in Alzheimer’s Disease and Other Dementias: Caregiver Awareness and Communication Strategies

**DOI:** 10.3389/fpsyg.2021.790025

**Published:** 2021-12-07

**Authors:** Alison Warren

**Affiliations:** The Department of Clinical Research and Leadership, The George Washington University, Washington, DC, United States

**Keywords:** Alzheimer’s disease, dementia, consciousness, caregivers, policy

## Abstract

Alzheimer’s disease is an insidious onset neurodegenerative syndrome without effective treatment or cure. It is rapidly becoming a global health crisis that is overwhelming healthcare, society, and individuals. The clinical nature of neurocognitive decline creates significant challenges in bidirectional communication between caregivers and persons with Alzheimer’s disease (AD) that can negatively impact quality-of-life. This paper sought to understand how and to what extent would awareness training about the levels of consciousness in AD influence the quality-of-life interactions in the caregiver-patient dyad. A literature review of multiple databases was conducted utilizing a transdisciplinary approach. The sum of findings indicates a positive relationship between enhanced caregiver awareness and training, positive interactions, and improved QOL measures among patients and caregivers. A multidirectional relationship was found among healthcare policies, training and education resources, caregivers, and persons with AD. Specifically, the current lack of policy and inadequate training and educational resources has various detrimental effects on patients and caregivers, while improvements in training and education of caregivers yields positive outcomes in communication and QOL. Furthermore, evidence of preserved consciousness in persons with AD was demonstrated from multiple disciplines, including neurobiological, psychological, and biopsychosocial models. The literature further revealed several methods to access the preserved consciousness in persons with AD and related dementias, including sensory, emotional, and cognitive stimulations. The evidence from the literature suggests a reframed approach to our understanding and treatment of persons with AD is not only warranted, but crucial to address the needs of those affected by AD.

## Introduction

Alzheimer’s disease (AD) is the most common diagnosis for individuals with dementia (U.S. Department of Health and Human Services, 2021). It is characterized as an insidious onset, age-related, neurodegenerative syndrome that ultimately results in death (Kumar et al., 2018). The most salient feature of AD is progressive memory decline, with associated language and other cognitive deficits, as well as psychological symptoms that may emerge in varying degrees (Stella et al., 2015; Leandrou et al., 2020). Like other dementias, AD exists on a continuum, ranging from preclinical stages to the more severe symptomatic late stages (Wadsworth et al., 2012; Ruiz-Gómez et al., 2021). Clinical manifestations follow a consistent pattern beginning with mild memory impairment, particularly episodic and autobiographical memories, and later extend to language and visuospatial deficits (Budson et al., 2016). In addition to neurocognitive domains, behavioral and psychological symptoms of dementia (BPSD) are another clinical feature of AD, and include symptoms such as depression, anxiety, apathy, and agitation (Peña-Casanova et al., 2012; Stella et al., 2015). It is postulated that the disease begins approximately 20 years before symptom onset (Caselli et al., 2020). When early signs and symptoms of decline do begin to manifest, they are often unnoticed until deficits begin to affect instrumental activities of daily living (IADLs), such as managing finances, medications, and household chores (Aretouli and Brandt, 2010), and finally basic activities of daily living (ADLs), such as bathing, dressing, feeding, and toileting will ultimately necessitate aid (Amjad et al., 2018).

Despite recent advances in sophisticated biomarkers, autopsy remains the most definitive diagnosis (King et al., 2020). Diagnosis is based on a combination of cognitive and memory testing; brain imaging, particularly PET to detect increased β-amyloid and MRI to detect atrophy; and lumbar puncture identify β-amyloid and tau in the cerebrospinal fluid (CSF) (Bird et al., 2010; Clarke et al., 2018; Alzheimer’s Association, 2020). To date, only the recently approved anti-amyloid monoclonal antibody, Aducanumab, has showed potential to treat AD, and requires more time to determine efficacy (Thomas et al., 2021). Despite its controversy, no other pharmacological treatments have demonstrated efficacy to slow or stop the neuronal destruction in AD (Alzheimer’s Association, 2020). While a cure remains elusive, some drugs have shown modest benefit in the temporary improvement of cognitive symptoms, such as the acetylcholinesterase inhibitors rivastigmine, galantamine, donepezil, and huperzine, and the NMDA receptor antagonist memantine (Kumar et al., 2018). The lack of disease-modifying interventions thereby emphasizes a clinical focus on non-pharmacological approaches to promote quality of life (QOL) in those living with AD (Faieta et al., 2021).

The conscious experience of those with AD is malleable throughout the disease process, and “even with widespread neurodegeneration, patients remain conscious and sentient” (Gazzaniga et al., 2019, p. 639). The constructs of internal representation, feelings, and sentience are foundations of consciousness (Damasio, 2012). For the purposes of this paper, the terms “consciousness” and “cognitions” will be used interchangeably, denoting the presence of thoughts, feelings, emotions, and perceptions. Although from a neurological point of view, “Levels of consciousness” can have other meanings such as alertness, for the purposes of this paper, they are both vertical and horizontal microcosms of our internal world. In other words, the various constructs may increase or decrease, but also change across a dynamic dimension based on intrinsic and extrinsic factors. As memory and cognition wax and wane in AD, the levels of consciousness, and sense of self can vary concomitantly, but are maintained (Sabat, 2018). In fact, despite emerging abnormalities, aspects of memory, cognition, emotion, and perception *are* preserved in this population, a fact that is poorly understood by many (Sabat, 2018; Gazzaniga et al., 2019). This implies that persons with AD may experience more cognitions and emotions, for example, than have the ability to effectively convey to caregivers and family (Swall et al., 2017).

The rich internal world of AD patients may be difficult for caregivers to discern or appreciate. However, understanding the subjective experience of AD patients can facilitate interpretation of behavior and influence quality of life (QOL) (Zwijsen et al., 2016). As AD worsens, caregivers often struggle with the interpretation of AD behaviors and communications, as well as strategies for reciprocal communication, creating a situation of mutual frustration within this dyad (Kim et al., 2021). This lack of awareness and understanding can create obstacles in communication and make the process of caring for those with AD even more challenging (Wilson et al., 2012; Pleasant et al., 2020). Recent data showing the lack of accessibility to caregiver education (Peterson et al., 2016) suggests the need for future policies surrounding the management of AD to prioritize caregiver training in awareness and communication skills as a standard of care.

## Methodology

This is a review of the literature utilizing a transdisciplinary lens to identify the preserved consciousness in persons with AD and dementia; as well as the potential of caregiver awareness and training to improve QOL and positive interactions. ADRD encompass a multifaceted interaction of physical, emotional, and cognitive constructs among and between the caregivers and patients. As such, a transdisciplinary lens through which to view persons affected by ADRD is critical in this regard. A search limited to peer-reviewed articles was conducted up to and including October 2021 through the Harvard Library Catalogue (HOLLIS), the George Washington University Health Sciences Library Catalogue (Himmelfarb). Google Scholar, PubMed, and PsychINFO databases. Topics included Alzheimer’s disease; dementia; neurology; neuroanatomy; neurobiology; gerontology; aging; consciousness; formal and informal caregivers; caregiver education and training; and education. The main exclusion criteria included more specific types of dementia with differing characteristic pathologies+, such as Vascular Dementia, Frontotemporal Dementia, Lewy Body Dementia, Parkinson’s Disease Dementia, and Korsakoff’s syndrome. The main keywords chosen in this search included “Alzheimer’s disease” and “dementia”; and the following inclusive AND keywords “consciousness”; “cognitions”; “caregiver education”; “caregiver training”; “music”; “exercise”; “communication”; “policy”; “quality of life.” In addition to searching the above-mentioned databases, snowballing techniques were used frequently in several seminal and innovative papers related to topic.

## The Current Status

As the most common form of dementia, Alzheimer’s disease (AD) affects approximately 6 million individuals aged 65 and older in the United States, with a projection of 13.8 million Americans expected to be diagnosed by mid-century (U.S. Department of Health and Human Services, 2019; Alzheimer’s Association, 2020). As AD continues to overwhelm the world as major health crisis, policy reformations are beginning to emerge to prioritize long-term care and support of this patient population and their associated caregivers (Ohno et al., 2021). Due to shortcomings in detection and underreporting by patients and families, the actual prevalence is likely much higher as AD is underdiagnosed (Amjad et al., 2018). In fact, it is estimated that only about half of individuals who meet diagnostic criteria are diagnosed by a clinician (Lang et al., 2017; Amjad et al., 2018). Worldwide, dementia has become a global health priority, as evidenced by the prevalence of AD as over 24 million and projected to double by the year 2040 (Adams, 2020; Grande et al., 2020). AD is the sixth leading cause of death in the United States and the total cost estimate for health care, long-term care, and hospice related expenses is $305 billion in the United States alone (Alzheimer’s Association, 2020). While the size of United States and global populations of individuals directly and indirectly affected by this disease continues to rapidly increase, the overwhelming burden on individuals and society will necessitate a more focused innovated policy approach to better serve this population.

Alzheimer’s disease is profoundly devastating to those experiencing and witnessing the disease progression (Rosin et al., 2020). The nature of Alzheimer’s disease is such that autonomy will gradually decline as AD patients demonstrate increasing difficulty with ADLs, leading to the inevitability of reliance upon a caregiver to perform basic tasks, maintain safety, and to carryout fundamental requirements for life (Cabinio et al., 2018).

The nature of AD BPSD can create significant challenges for the most well-intentioned caregivers (Honda et al., 2018). Lack of education and awareness of the origins of this behavior and affect can result in considerable psychological and physical detriment to all parties (Wang et al., 2018). The care provided by caregivers, along with difficulties in communication, can create considerable physical and emotional strain (Isik et al., 2019). This dyad may therefore experience substantial distress with consequent reductions in quality of life (Isik et al., 2019).

Furthermore, it was found that obtaining educational materials can prove difficult for caregivers, and information and training resources are often inadequate or unoffered entirely (Peterson et al., 2016). Professional caregivers in long-term care facilities are likewise undertrained in the neurocognitive and affective aspects of AD (Whitlatch and Orsulic-Jeras, 2018). Knowledge and training in communication skills with this unique patient population is often commensurately insufficient (Conway and Chenery, 2016). Systematic policies to implement such strategies are lacking in this regard (Stites et al., 2018). Furthermore, recent estimates have shown that the medical field in the United States only has about half of the geriatricians it needs to meet the demands of our rapidly growing population (Peterson et al., 2016). This lack of skillset extends to family and caregivers, who receive little or no education regarding the pathology, nor training in communication skills (Eggenberger et al., 2013).

Currently, many recommendations have been theorized, but a widespread policy change not yet been implemented at this juncture (Pleasant et al., 2020). Considering the dramatic rise in this patient population, and increasingly pervasive effects in multiple aspects of society, this major health crisis demands reform to yield more positive outcomes (Olivari et al., 2020). Taken together, this suggests that any “gold-standard” of care should involve awareness of the relationship between the patient’s conscious experience, their behaviors, and the resulting impact on QOL within this dyad. An initial survey of the literature on “best practices” in AD care revealed little evidence, however, that individuals are informed about consciousness in AD.

Considering the global magnitude of those affected by this disease, a multidisciplinary, person-centered approach to understanding the neurocognitive, psychosocial, and educational underpinnings as related to preserved consciousness in AD will be examined. In the same vein, this paper will also explore avenues with which medical professionals, patients, and caregivers can access and apply information to thereby facilitate positive interactions and increase QOL parameters.

## The Problem

The ever-growing population of persons with Alzheimer’s disease and related dementias (ADRD) is overwhelming the world in several aspects, from the individual level to society. The need for high quality caregiving is likewise escalating concomitantly. After diagnosis, persons with AD typically live an average of 4–8 years, and some up to 20 years (Kokorelias et al., 2020). The shortage of medical specialists for ADRD places a great burden on primary care providers, who feel ill-equipped and undertrained to advise or treat this patient population (Peterson et al., 2016; Bernstein et al., 2019; Kistler et al., 2020). Consequently, many family members and caregivers receive little or no education or training in communication strategies (Peterson et al., 2016), nor information regarding the facets of consciousness that are preserved in persons with AD (Sabat, 2018). Yet, knowledge of the internal world of persons with ADRD can increase understanding of behaviors and experiences, thereby facilitating communication (Sabat, 2018). The lack of such resources and training, along with a cohesive policy approach, represents a significant gap in literature and practice.

## Clinical Manifestations of Alzheimer’s Disease

The risk and protective factors that contribute to the development or prevention of AD are vast. The multifactorial milieu of this disease reflects the complex interplay between genetics and environment. The main risk factors that have been identified include the APOE gene; vascular diseases including hypertension; cerebrovascular disease (CVD); Type 2 Diabetes Mellitus; epilepsy; psychological illness such as depression; traumatic brain injury; and lifestyle factors such as excessive alcohol use; tobacco; sedentary lifestyle; poor diet; and disturbed sleep (Edwards et al., 2019). Protective factors include many modifiable lifestyle habits including diet, especially a Mediterranean diet (Edwards et al., 2019; Grande et al., 2020); alcohol in moderation (Edwards et al., 2019; Grande et al., 2020), particularly red wine (Edwards et al., 2019); physical activity (Edwards et al., 2019; Grande et al., 2020); adequate sleep (Edwards et al., 2019); education (Grande et al., 2020); and life-long engagement in cognitively and socially stimulating activities (Grande et al., 2020).

Persons with AD exhibit a multitude of cognitive, affective, and physical manifestations to variable degrees at different stages. Aphasia can manifest as an early feature in some patients, particularly word-finding difficulty and circumlocution; apraxia and visuospatial deficits then typically follow but vary considerably in time of onset (Bird et al., 2010; Ota et al., 2020). Higher brain functions, including executive functions, continue to decline as the disease progresses, such as apraxia, aphasia, and agnosia; and reading impairment manifests as atrophy reaches the posterior cortex (Ota et al., 2020).

It is important to note that many presentations of AD pathology do not necessarily follow a “typical” course, but rather exhibit a variety of presenting symptoms consistent with location of pathology. AD typically originates in the entorhinal cortex and spreads to the hippocampus, amygdala, and the posterior temporal and parietal cortices, and eventually progresses to diffuse degeneration in the cortex (Leandrou et al., 2020; Olajide et al., 2021). Recently, it has been proposed that subtypes of AD exist, accounting for variability in disease distribution and manifestation.

Hippocampal-sparing AD is associated with earlier age of onset, a more rapid rate of cognitive decline, and atypical clinical presentations in 30% of cases (DeTure and Dickson, 2019). Hippocampal-sparing AD may also present similar to patients with primary progressive aphasia as evidenced by agrammatic, semantic, or logopenic presentations (DeTure and Dickson, 2019). Posterior cortical atrophy (PCA), sometimes associated with hippocampal-sparing AD (DeTure and Dickson, 2019), generally has a younger age of onset and is “characterized by a progressive decline in visuospatial, visuoperceptual, literacy, and praxic skills (Crutch et al., 2012, p. 170). This “visual dementia” results in disintegration of the visual world for these persons but distinguished from the more amnestic presentations commonly seen in persons with dementia (Crutch et al., 2016). Limbic-predominant AD is associated with later age of onset and slower progression (DeTure and Dickson, 2019). Further possible subtypes include minimal atrophy type (Ferreira et al., 2020) and capillary cerebral amyloid angiopathy type, the latter of which is linked to the APE4 and a variant in the receptor for apolipoproteins (LDL receptor related protein 1) (DeTure and Dickson, 2019).

Neuropsychiatric symptoms are often expressed later in the disease but can occur at any stage. BPSD can include depression, anxiety, and apathy in earlier stages, and often progress to irritability, agitation, and delusions as the disease worsens (Peña-Casanova et al., 2012). Disruptions in sleep-wake patterns and gait begin to emerge as well, and for those who remain ambulatory often display a tendency to wander (Bird et al., 2010). As neuronal death becomes more severe, basic bodily functions are impaired necessitating total dependence for care (Alzheimer’s Association, 2020). Death often results from malnutrition, secondary infections, or such severe neuronal deterioration that is incompatible with life (Bird et al., 2010; Alzheimer’s Association, 2020).

## Consciousness in Alzheimer’s Disease: Preserved Cognitions, Emotions, and Subjective Experience

Understanding the true extent and capacity of subjective experience in persons with AD and dementia may aid in the amelioration of the depersonalization and multiple threats to self and personhood sustained by these individuals (Yatczak, 2018). The word “dementia” itself denotes removal of the mind in its Latin roots, thereby perpetuating misconceptions that persons with dementia suffer a loss of personality, of mind, and of self (Halewood, 2016). Thus, a prioritization of terminology and its potential impact on malignant social psychology is essential (Lenzoni et al., 2020).

The definitions, constructs, and neural correlates of consciousness have been explored by many in the fields of philosophy (e.g., Plato, Aristotle, Descartes, John Locke, Jean-Paul Sartre, Immanuel Kant, Ned Block, and Thomas Nagel), psychology (e.g., William James, William Wundt, and B. F. Skinner), medicine (e.g., Zeki and Lamme, Voss, Schiff, Jennett and Plum, and Chalmers), and medical neuroscience (e.g., Stanislas Dehaene, Antonio Damasio, Michael Gazzaniga, Francis Crick, Christof Koch, Oliver Sacks, and V. S. Ramachandran). The definition offered by Stuart Sutherland (1989) is most apropos in this regard. Sutherland defines consciousness as “the having of perceptions, thoughts, and feelings; awareness,” yet still impossible to define (Sutherland, 1996, p. 95). Consciousness and cognition become variably aberrant in patients with AD (Salmon et al., 2005), but quite notably, are not abolished (Moustafa and El Haj, 2018; Sabat, 2019). Additionally, while many patients display anosognosia (lack of awareness regarding their deficit), others display considerable awareness about their deficits (Bird et al., 2010). Even in those with evident anosognosia, several studies suggest that while self-consciousness is altered, it is anything but absent (Arroyo-Anlló et al., 2020). Despite the challenges and barriers to education and communication strategies, it has been established that dementia and AD patients *do* retain a sense of self, consciousness, memory (Arroyo-Anlló et al., 2020), social sensitivity, and emotion (Fredericks et al., 2018).

### Memory and Awareness in Persons With Alzheimer’s Disease

Anosognosia, a form of metacognitive deficit, may vary in time of onset, but tends to worsen as AD progresses (Geurten et al., 2021). Studies have demonstrated preserved implicit metacognition in persons with AD (e.g., Golby et al., 2005; Sabat, 2006; Deason et al., 2019; Geurten et al., 2021), although explicit metacognition becomes increasingly impaired. The relevant distinction, in this regard, is the ability to *explicitly* consciously recall or recognize, as opposed to *implicitly* coding and retrieving without conscious awareness, as evidenced by a change in behavior or performance as result of previous experience or exposure (Sabat, 2006). A widely used example in the literature is the word-stem completion task, in which a subject is presented with a list of words to review. When subsequently asked to state the first word that comes to mind with the stem (i.e., “def-”), AD (and other cognitively compromised) subjects will recall the correct word from the list (i.e., “defend”), without recalling that they were ever shown a list (Sabat, 2006; Teresa Redondo et al., 2015). In this way, they cannot explicitly recall reviewing the list, but they do implicitly display evidence of memory of the words to which they were exposed.

This concept of preserved implicit memory is highly pertinent and actionable, but often overlooked. An article by Sabat (2006) elucidates the scant knowledge of implicit memory among professionals and caregivers who fail to delineate recall from memory formation. He emphasizes the “remarkable frequency” with which they assume that “defects in the ability to recall the details of recent events and experiences means that the person with AD has no memory of those recent events and experiences” (Sabat, 2006, p. 11). Nonetheless, past fMRI studies of AD patients have demonstrated impaired explicit recognition memory, but intact implicit memory (Golby et al., 2005). Recent studies have elucidated neurobiological compensatory mechanisms of preserved implicit memory (i.e., left-lateralized memory circuit dropout and right hemisphere compensation) that account for a range of performance metrics (Han et al., 2007; Tyrer et al., 2020).

Autobiographical memory, which triggers a state of autonoetic consciousness, becomes increasingly compromised in AD (El Haj et al., 2019). A recent study by El Haj et al. (2019) investigated whether autobiographical retrieval would improve a sense of self in AD. Compared to controls, AD patients demonstrated better production of descriptions related to physical, social, and psychological self after autobiographical retrieval, suggesting the potential value to activate the sense of self in persons with AD. Additionally, spaced retrieval memory training studies have demonstrated the ability of AD patients to learn and recall new episodic information, which can improve short and long-term recall (Small and Cochrane, 2020). Furthermore, there is evidence to support that certain aspects of selfhood, such as bodily awareness and social interaction (e.g., interpersonal self) remain preserved even in the final stages of the disease (Lenzoni et al., 2020). The manner in which a person with AD is treated by healthy others affects their sense of self greatly, reflecting the “concept of *ubuntu*, that a person is a person through others” (Sabat, 2009, p. 24).

A phenomenological corollary to the concept of consciousness is illustrated by instances of paradoxical lucidity (PL) in AD and dementia patients. While not exclusive to AD, these episodes of spontaneous, meaningful communication and connectedness in individuals presumed to have lost the capacity for coherent verbal and behavioral interaction challenge over a century of assumptions (Mashour et al., 2019). These cases of memory retrieval, often near death, bring into question the central “irreversible” paradigm of dementia, thereby supporting the construct of dementia as a problem with memory retrieval rather than memory consolidation (Bostanciklioğlu, 2021).

The mechanisms of the network level return of PL are currently under exploration, hypothesizing the fluctuation of brainstem neuromodulator circuitry projecting to the medial prefrontal cortex and hippocampus, in addition to corticotropin-releasing peptides that may increase mental clarity and excitability of this circuitry (Mashour et al., 2019; Bostanciklioğlu, 2021). A recent study of 124 dementia patients who exhibited pre-death PL suggested the existence of a reversible and functional aspect of pathophysiology of dementia, even in severe cases (Batthyány and Greyson, 2020). Consistent with other available studies, these patients died in close proximity to the PL episode. Observers reported complete remission characterized by a return of memory, orientation, and verbal responsiveness in over 80% of cases (Batthyány and Greyson, 2020). While our current understanding is such that degenerative dementias are irreversible and some caution should be reserved with caregiver reports, these remarkable occurrences are providing new insights not only in AD pathology, but brain physiology as well.

### Emotions in Alzheimer’s Disease

The emotional component of the AD consciousness continuum is poignantly framed by Fredericks et al. (2018). They state that AD patients commonly display “strikingly preserved social sensitivity. They retain the emotional trace of film clips despite forgetting their narrative content, show preserved mutual gaze with their spouses, and have a heightened tendency to take on the emotions of those around them,” which, when negative, can manifest as BPSD (Fredericks et al., 2018, p. 471). As verbal communication abilities worsen in persons living with AD, emotions remain a key element of communication, and are preserved even into the late stages of the disease (Lee et al., 2019a). Even in later stages, while persons with AD may not have the capacity to recall the source of their emotional experience, their self-reported daily emotion retained validity and reliability (Zhang et al., 2015). Emotional expression signifies underlying needs and feelings, the recognition of which is crucial to implementation of person-centered care (Lee et al., 2019a). In fact, optimizing preserved emotional capacities may “help AD patients to compensate for, and resiliently adapt to the impairment of cognitive functions caused by the disease” (Zhang et al., 2015, p. 208).

### Non-pharmacological Interventions to Affect Consciousness and Quality of Life

Methods used to stimulate consciousness have been used in throughout the ages. The use of sensory stimulations to access memory, consciousness, emotions, and self in patients with neurological disorders is exemplified by the late neurologist Oliver Sacks. Sacks’ case histories and intervention methods, particularly in his books *Awakenings* and *Musicophilia*, are era-defining, and have resulted in expansions in perspectives and treatment in patients with neurological disorders, including AD (Sacks, 1990, 2008).

Modes of sensory and motor stimulation have been explored as non-pharmacological approaches to affect consciousness and enhance quality of life in patients with dementia, including exercise (Hoffmann et al., 2016; Kishita et al., 2020; Teri et al., 2020), music (Murphy et al., 2018; Kishita et al., 2020), cognitive behavioral therapy (CBT) (Kishita et al., 2020) psychosocial intervention (Kishita et al., 2020; Teri et al., 2020), and art therapy (Murphy et al., 2018). Kishita et al. (2020) conducted an overview of systematic reviews of such interventions to improve QOL in people with dementia and found effective interventions to include cognitive stimulation, psychological treatments such as CBT, and music-based interventions. As QOL can be difficult to quantify, they also concluded that Lawton’s model of QOL, “which emphasizes multiple overarching dimensions that contribute to QoL” such as psychological well-being, behavioral competence, and objective environment (including caretakers), “has undoubtedly been the most pervasive influence on conceptualizations of QoL and the development of QoL instruments for this population” (Kishita et al., 2020, p. 28). However, it is important to note that a clear, shared definition of QOL in persons with dementia is still absent (Dourado et al., 2020) and ratings are often influenced by carer’s own level of burden (Kishita et al., 2020).

The effects of exercise implementation in AD have long been established. A 2004 meta-analysis by Heyn et al. (2004) concluded that exercise increases physical and cognitive function, as well as positive behavior in dementia patients. A 2015 systematic review and meta-analysis of randomized controlled trials (RTCs) examining the effects of exercise on BPSD found reduced levels of depression, but not global BPSD (de Souto Barreto et al., 2015). A cross-sectional study by Sampaio et al. (2020) found that physical fitness positively affected QOL in institutionalized individuals with dementia, with positive implications for cognitive function and functional capacity. A later RCT examined mild to moderate intensity exercise in AD patients and found exercise reduced neuropsychiatric symptoms, with an additional beneficial finding of possible preserved cognition (Hoffmann et al., 2016).

There is a growing interest in exploring the potential to utilize sensory perceptions to facilitate positive emotions and interactions in AD patients. Newer studies suggest that emotional sensorial stimulations and multisensorial stimulations in AD patients that include odor (e.g., El Haj et al., 2018), music (e.g., Gulliver et al., 2021), taste (mealtime interventions) (e.g., Smith and D’Amico, 2020), and healing gardens (e.g., Uwajeh et al., 2019) have a positive impact on self-consciousness (SC); elicit autobiographical memories; can temporarily exalt memory, affective state, and personal identity; and could therefore serve to improve QOL in patients and caregivers (Arroyo-Anlló et al., 2020). It is worth noting that other studies suggest that rich sensory stimulation alone is insufficient, highlighting the importance of the emotional component of the sensorial stimuli as the key element that can enhance cognitive, affective, and behavioral components of the patient’s well-being (Arroyo-Anlló et al., 2020). With emotion as a focus, Arroyo-Anlló and Gil (2020) poignantly stated:We focus on something essential in life that can be stimulated by daily activities such as smell, taste, and music, because although the patient is unable to identify/name the smell, dish, or musical piece, they can experience emotions, relive situations, find the emotions that contribute to and recognize it. From this perspective of “care,” neuropsychology also has an essential role to play, since it can scientifically prove that even temporarily, certain protocols exalt memory, their affective state, and their personal identity—that is, their SC (Martin et al., 2020, p. 593).

In this way, one could ascertain that emotion and self-consciousness are inextricably tied, and self-consciousness is comprised of, and accessed by, several different constructs.

In AD, brain areas involved in music processing are preferentially spared, allowing for recognition, heightened arousal, improved attention and memory, and reduced BPSD (Simmons-Stern et al., 2010; Buller et al., 2019). Musical stimulation that is familiar may potentially serve as an enhancer of self-consciousness in AD patients (Arroyo-Anlló et al., 2013), supporting further findings of implicit compared to explicit memory for which AD patients display preference, and rely heavily upon information that is familiar (Deason et al., 2012). As Oliver Sacks explained in his book *Musicophilia*, for patients with neurological disorders, music can provide access to movement, speech, and life, even when no medication can (Sacks, 2008).

As an extension to this idea, personalized music listening (PML) has arrived at the forefront of AD musical intervention by a non-profit group, Music and Memory, led by Dan Cohen, responsible for the award-winning documentary *Alive Inside* (Murphy et al., 2018). Personalization of music is posited to “provide autobiographical context that connects the listener to remote memories, emotions, and verbal abilities preserved despite the neurocognitive decline” (Murphy et al., 2018, p. 3). The Alzheimer’s Association of Central and Western Kansas recently implemented “The Rother Project – Music and Memory,” and found that personalized music elicited improvements in mood, overall happiness, emotional expression, and decreases in anxiety and depression (Buller et al., 2019). It is important to note that in a study by Garrido et al. (2019), AD patients with high levels of depression demonstrated increased sadness while listening to music in minor keys, while AD patients with low level depression demonstrated the highest levels of pleasure with music intervention. Overall, music with fast tempos increased arousal and enjoyment, yet manifestations of variable reactions in depressed patients suggests the importance of considering mental health as part of the individualization of intervention (Garrido et al., 2019).

Finally, another strategy to enhance personhood and QOL involves animal-assisted therapy (Swall et al., 2017). Studies involving therapy animals have shown positive effects. An example is a study by Swall et al. (2017) examining interactions of persons with AD and a therapy dog demonstrated an extension of social interaction that elicited feelings of joy, altruism, tenderness, and empathy that lend to feelings of meaningful experience, being needed, and self-worth.

## Caregivers

The combined loss of autonomy for the patient and transition to caregiver role for family can sustain a chronic stress response, thereby increasing the risk of cognitive decline for *both* parties (Corrêa et al., 2019). Many patients remain at home with a spouse or family member during the early stages of the disease, and later transition to long-term care (LTC) facilities as IADLs and ADLs become compromised (Alzheimer’s Association, 2020). Studies estimate prevalence of cognitive impairment or ADRD patients in assisted living facilities to range from 40 to 72% (Thomas et al., 2020).

When persons with AD remain at home, the informal caregiver (i.e., family members) sustain increasing responsibility and challenges in caring for the basic needs of their loved ones (Lee et al., 2019b). These caregivers have “unmet needs for general information regarding Alzheimer’s disease, tangible care services, respite, and emotional and financial support, and learning skills for improving daily care management” (Kokorelias et al., 2020, p. 2). Females in particular seem to experience more family conflict, depression, and distress compared to male during the time of transition to caregiver role (Lee et al., 2019b). A recent study of informal caregivers and persons with dementia identified the most commonly unmet needs encompassed home and personal safety (e.g., medication use), general health care (e.g., dental care), and daily activity domains (e.g., physical activity and social isolation) (Black et al., 2019). Social, professional, psychosocial, education, and behavioral interventions have shown inconsistency in effectiveness (Kokorelias et al., 2020). Transition to the caregiver role necessitates AD specific information, emotional support that also involves peer support, and assistance planning care (Lee et al., 2019b). To address the dynamic nature of AD, a phasic approach to address caregiver needs is an important consideration in managing the fluctuations and severity progression that occur over time. A study of caregiver needs by Kokorelias et al. (2020) identified 5 categories of needs across the caregiving phases, including monitoring initial symptoms, navigating diagnosis, assisting with IADLs, assisting with basic ADLs, and preparing for the future. Of note, the Scottish government has enacted strategies dedicated to providing support for family caregivers and persons with dementia (The Scottish Government, 2017). Their program provides families with education and support for 1 year following AD diagnosis and longer if needed (The Scottish Government, 2017; Lee et al., 2019b).

### Communication Difficulties and Repercussions in the Caregiver-Patient Dyad

The characteristics of AD pathology can create considerable difficulties in communication between the patient and caregiver. As demands of care increase, many caregivers experience a substantial amount of stress as understanding and communications commensurately degrade (Nguyen et al., 2019). This can lead to significant detriment to all parties involved; the emotional, physical, and financial tolls of which often result in depression, burnout, reduced quality of life, and suicides (Nguyen et al., 2019; Kim et al., 2021). In addition to the strain experienced by caregivers, their distress often impacts the person with dementia as well, creating a cycle of distress, misunderstanding, and deterioration of relationships (Savundranayagam and Orange, 2014). This cycle can perpetuate distress between and within each member of the relationship.

The resulting psychological and physical tolls exacted on patients and caregivers are considerable. Indeed, caregiver distress has been associated with increases in BPSD, patient abuse, and odds of institutionalization of the dementia patient (Peterson et al., 2016). In both patients and caregivers, negative outcomes such as general health decline, decreased QOL, and increased risk for morbidities have been associated with caregiver burden (Isik et al., 2019). Evidence has demonstrated a bidirectional relationship between caregiver burden and an increase in neuropsychiatric symptoms in AD patients (Peterson et al., 2016; Isik et al., 2019). This feedback mechanism often results not only in a worsened relationship between caregiver and patient, but an increase in family conflict as well (Isik et al., 2019; Kim et al., 2021).

Considering the widespread communication breakdown inherent in this disease process, relaying this information to professionals and caregivers is of paramount import (Wilson et al., 2013; Savundranayagam and Orange, 2014). Understanding the extent to which consciousness is preserved in this patient population holds the potential to not only facilitate positive interactions, but also increase QOL in patients and caregivers (Conway and Chenery, 2016). For example, the ability to recognize emotional responses allows caregivers to assess feelings and needs “(e.g., unmanaged pain, hunger, and thirst) and care preferences that are fundamental to implementing person-centered care” (Lee et al., 2019a, p. 345). Along these lines, Sabat (2019) asserts that “understanding the cognitive and social strengths of people diagnosed is of paramount importance for developing optimal caregiving approaches” (Sabat, 2019, p. 163).

### Person-Centered Care

In the early 1980’s, Kitwood (1988, 1993) pioneered a seminal paradigm shift to create a more ethnocentric approach to dementia research and person-centered care (PCC) within this particular patient population (Wu et al., 2020). In contrast to the pervasive misconception in which this population suffers an inexorable loss of self (Halewood, 2016), this approach reframes the individual with dementia “as a person in the fullest possible sense: he or she is still an agent, one who can make things happen in the world, a sentient, relational and historical being” (Kitwood, 1993, p. 541).

Kitwood (1993, p. 542) coined the term “malignant social psychology” (MSP) to denote behaviors that individuals may perform, without intentional malice, toward patients with dementia; the effects of which are “highly damaging from the recipient’s point of view” (e.g., treachery; disempowerment; infantilization; condemnation; intimidation; stigmatization; outpacing; invalidation; banishment; and objectification). He also operationalized twelve indicators of well-being, highlighting preserved emotions and cognitions of this population, including “assertion of desire, emotional ambience, initiation of social contact, showing affection, sensitivity to others’ feelings, self-respect, acceptance of other dementia sufferers, humor, creativity, helpfulness, taking pleasure, and physical relaxation” (Kitwood, 1993).

Terkelsen et al. (2020) reviewed 154 studies investigating the experiences of dementia patients and formal caregivers’ use of Kitwood’s framework in institutional settings. 19 articles were ultimately included, comprised of predominantly peer-reviewed articles, followed by dissertations, conference posters, and non-peer-reviewed articles from 1998 to 2016 (Terkelsen et al., 2020). The authors found overall positive experiences gained from applying this approach to PCC in clinical practice.

A systematic review and meta-analysis of PCC effectiveness for PWD was conducted by Kim and Park (2017). 19 intervention studies involving 3, 985 participants were included and likewise determined that PCC interventions significantly improved QOL in persons living with dementia. Finally, a systematic review including articles up to June 2018 was conducted by Chenoweth et al. (2019) to determine the efficacy of PCC at the organizational level. Their results of 12 eligible studies involving 2599 PWD yielded significant results for QOL effect, and thus a recommendation to implement PCC at the organizational level to support QOL in PWD (Chenoweth et al., 2019).

## Policy

It has been estimated that more than 80% of caregivers report the need for more information regarding caregiver topics (Peterson et al., 2016). In 2016 it was estimated by the Older Americans Act’s National Family Caregiver Support Program that over half of Area Agencies on Aging “did not offer evidenced-based family caregiver interventions” (Alzheimer’s Association, 2020, p. 419). While some resources are available, they are seldom readily accessible or provided, thereby placing the burden of searching for educational resources on the family and caregivers, illustrating a growing need in this regard (Whitlatch and Orsulic-Jeras, 2018). As such, we do not yet know how the integration of this knowledge to caregivers as part of the treatment plan could affect quality of life for those affected by AD and other dementias.

Furthermore, in the setting of care facilities, turnover rates remain high among care workers who receive inadequate education in these challenging work environments, which has also been associated with poorer quality of care (Alzheimer’s Association, 2020; Dassel et al., 2020). Research has shown that caregivers and staff who have the least amount of training and support demonstrate the highest levels of stress and burnout (Pitfield et al., 2011; Costello et al., 2019; Dassel et al., 2020). As the demand for caregivers increases, the various barriers persist proportionately. Direct care of nursing home residents is largely provided by certified nursing assistants (CNAs), the majority of whom have no more than a high school level education and make little more than minimum wage (Squillace et al., 2009). The combination of a patient population who have difficulty articulating their needs, caregivers who are not adequately trained in interpreting nor communicating with them, in the setting of a labor-intensive environment is an impediment to QOL in all parties (Nguyen et al., 2019; Pleasant et al., 2020).

However, there is emerging evidence that implementation of training programs and educational resources have positive impacts within this dyad. A systematic review by Nguyen et al. (2019) concluded that although more research regarding educational interventions is needed, there was “solid evidence for positive impact of communication training on the skills and knowledge of carers” (Nguyen et al., 2019, p. 1050). A randomized controlled trial by Birkenhäger-Gillesse et al. (2020) examined the effects of dementia training in caregiver-patient dyads via “The More at Home with Dementia intervention.” While no significant difference was found in care-related QOL, positive secondary outcomes were identified (e.g., better acceptance and coping, improved knowledge of dementia, community services and facilities, less pain, and positive effects on physical and emotional role limitations) (Birkenhäger-Gillesse et al., 2020). Dassel et al. (2020) developed an online, asynchronous training module focused on ADRD designed to improve best care practices in LTC settings. Their data revealed that “even just a few hours of additional specialized training in ADRD care practices improves knowledge of best care practice in LTC settings” (Dassel et al., 2020, p. 153).

Targeted training programs are also emerging to address well-being and QOL in persons affected by ADRD. Specific training programs utilizing Kitwood’s framework of person-centered dementia care have been expounded upon by numerous researchers in attempts to implement the principles critical for success into practice. A recent review article evaluated several of the components of these models, [e.g., Brooker (2003) VIPS model; Edvardsson et al. (2008) six components PCC; Love and Pinkowitz (2013) four component model; and the relationship-centered care models by Nolan et al. (2004) and Adams and Gardiner (2005), and identified three main positive outcomes of PCC within the dyad, (1) social well-being; (2) psychological well-being; and (3) physical well-being (Wu et al., 2020)]. While promising, there remains a lack of research on person-centered care implementation into nursing home care (Boersma et al., 2019). A newer PCC method, the Veder contact method (VCM), was studied in the setting of a 24-h residential care facility by Boersma et al. (2019). They found significant improvements in caregivers’ communicative behavior and positive interactions, as well as improvements in resident’s behavior and QOL from this emotion-oriented care model (Boersma et al., 2019).

Although many hundreds of care interventions have been tested in RCTs, the evidence needed to inform decisions about policy and actionable intervention is lacking (Larson and Stroud, 2021). The National Academies of Sciences, Engineering, Medicine, and Committee on Care Interventions for Individuals with Dementia Their Caregivers issuing body (2018) concluded that despite the lack of interventions that have met evidential criteria, there is sufficient evidence to justify implementation of collaborate care models and resources for enhancing Alzheimer Caregiver Health (REACH) models (National Academies of Sciences, Engineering, Medicine, and Committee on Care Interventions for Individuals with Dementia Their Caregivers issuing body, 2018; Larson and Stroud, 2021).

Finally, since the 2007 Healthy Brain Initiative (HBI) roadmap, a recent iteration of the Road Map was released in 2018, The HBIs *State and Local Public Health Partnerships to Address Dementia: The 2018–2023 Road Map* (Olivari et al., 2020). This latest iteration “identifies 25 actions that state and local public health agencies and their partners can implement to promote cognitive health and address cognitive impairment and the needs of caregivers” (Olivari et al., 2020, p. 1). These actions are categorized into four areas including monitor and evaluate, education and empower the nation, develop policies and mobilize partnerships, assure a competent workforce (Alzheimer’s Association and Centers for Disease Control and Prevention, 2018; Centers For Disease Control Road map for state and local public health and Centers for Disease Control and Prevention, 2020; Olivari et al., 2020). “Progress will require collaboration across sectors and by state and local agencies to improve systems and enhance service delivery in each and every community” (Alzheimer’s Association and Centers for Disease Control and Prevention, 2018; Centers For Disease Control Road map for state and local public health and Centers for Disease Control and Prevention, 2020).

## Conclusion

The beautifully complex constructs of consciousness and personhood are paradoxically personified in persons with dementia and AD. Yet, our current paradigms continue to place a disproportionate emphasis on deficits rather than preserved faculties and constructs of consciousness apparent within persons with AD. Consequently, caregivers are not only burdened with navigating their own education and awareness to care for their loved ones and patients, but what little information they are able to access is lacking in this regard. Without such awareness and training, the depth of preserved relationships, positive interactions, and well-being is compromised for all those involved in this challenging process. There is a loss in translation, if you will. To say this is a loss of opportunity for persons affected by AD is not only a gross understatement, but an opportunistic environment for negative outcomes, including but not limited to, reduced quality of life, depression, negative health consequences, abuse, and early demise.

Despite established and emerging research supporting that enhanced caregiver education and awareness can facilitate positive interactions and potentially improve QOL for all parties, the reported insufficiency of said training suggests more research is needed to establish avenues for implementation. On the other hand, awareness of such constructs of consciousness could potentially serve as the backdrop to enhanced communication with dementia patients. Devoid of such education, misperceptions, stigmas, and lost opportunities for quality interactions may ensue. A paradigm shift in the way the world should best perceive the subjective experience of dementia patients, our perceptions and interactions, and dementia patients themselves is of critical import to disabuse long-standing preconceived notions, biases, stigmas surrounding this disease, and the persons struggling with this disease.

Additional research is warranted to determine QOL parameters in a patient population who routinely experience difficulty articulating their thoughts and feelings reliably. Further in-depth research is also warranted to establish best practices to implement caregiver awareness and training to improve upon these parameters.

### General Summary

This project began as an intuitive suspicion of preserved consciousness in persons with AD from personal and professional experience. The information gleaned from the literature was far more profound than anticipated. Evidence of preserved consciousness was found from multiple perspectives, including neurobiological, neurophysiological, psychological, and sociological paradigms. Evidence was supported by imaging, experiments, and anecdotes. This project also provided a reframing of the problem of caregiver awareness and training, in that, it is not only a lack of resources acting as an impediment, but even more so a lack of policy to implement resources that are available. Policy reformations are underway, but far from fulfilling the dire need of the current and impending populations affected by AD.

A lack of focus on the constructs that are preserved as a priority in formal medical education and informal caregiver training is severely disproportionate. However, the plethora of evidentiary literature creates a hopeful potential for shift in our understanding and treatment of persons with AD, as well as avenues for improved support for caregivers. Indeed, bringing the concept of preserved consciousness to the forefront could reframe the focus of this disease to emphasize personhood and quality of life. The extraordinary examples of paradoxical lucidity have profoundly shaken the current understanding of consciousness and neuropathology in AD, dispelling the myth of a perpetual abyss in which we believe these patients to reside, thereby demanding a re-evaluation of our current knowledge. The psychological and interpersonal understandings of consciousness and communications strategies holds the potential to improve several QOL parameters in persona affected by AD, as well as our general perspective of persons with AD.

## Author Contributions

The author confirms being the sole contributor of this work and has approved it for publication.

## Conflict of Interest

The author declares that the research was conducted in the absence of any commercial or financial relationships that could be construed as a potential conflict of interest.

## Publisher’s Note

All claims expressed in this article are solely those of the authors and do not necessarily represent those of their affiliated organizations, or those of the publisher, the editors and the reviewers. Any product that may be evaluated in this article, or claim that may be made by its manufacturer, is not guaranteed or endorsed by the publisher.
